# Polarization and cell-fate decision facilitated by the adaptor Ste50p in *Saccharomyces cerevisiae*

**DOI:** 10.1371/journal.pone.0278614

**Published:** 2022-12-20

**Authors:** Nusrat Sharmeen, Chris Law, Cunle Wu

**Affiliations:** 1 Division of Experimental Medicine, Department of Medicine, McGill University, Montreal, Quebec, Canada; 2 Centre for Microscopy and Cellular Imaging, Department of Biology, Concordia University, Montreal, Quebec, Canada; 3 Human Health Therapeutics Research Centre, National Research Council Canada, Montreal, Quebec, Canada; Institute of Biology Valrose, FRANCE

## Abstract

In response to pheromone, many proteins localize on the plasma membrane of yeast cell to reform it into a polarized shmoo structure. The adaptor protein Ste50p, known as a pheromone signal enhancer critical for shmoo polarization, has never been explored systematically for its localization and function in the polarization process. Time-lapse single-cell imaging and quantitation shown here characterizes Ste50p involvement in the establishment of cell polarity. We found that Ste50p patches on the cell cortex mark the point of shmoo initiation, these patches could move, and remain associated with the growing shmoo tip in a pheromone concentration time-dependent manner until shmoo maturation. A Ste50p mutant impaired in patch localization suffers a delay in polarization. By quantitative analysis we show that polarization correlates with the rising levels of Ste50p, enabling rapid cell responses to pheromone that correspond to a critical level of Ste50p at the initial G1 phase. We exploited the quantitative differences in the pattern of Ste50p expression to correlate with the cell-cell phenotypic heterogeneity, showing Ste50p involvement in the cellular differentiation choice. Taken together, these findings present Ste50p to be part of the early shmoo development phase, suggesting that Ste50p may be involved with the polarisome in the initiation of polarization, and plays a role in regulating the polarized growth of shmoo during pheromone response.

## Introduction

Polarization is a directional growth of a cell in response to a stimulus, facilitated by localized organization of proteins through complex mechanisms, to orchestrate diverse cellular processes. Cell polarity exists in both prokaryotic and eukaryotic systems such as in bacterial chemotaxis, yeast mating and budding, in mammalian embryonic development, axonal guidance, and neutrophil migration in immune responses [1].

A well-studied and iconic cell polarization event occurs in yeast *Saccharomyces cerevisiae* when cells are exposed to the mating pheromone, shaping them into mating projections called shmoo. In the presence of both mating partners, *MAT*a and *MAT*α cells, shmoo develops directionally towards the opposite partner, using the pheromone gradient sensing mechanism, to ultimately fuse together [2]. The origin of this polarization event starts with the signaling branch of the pheromone response at a G-protein coupled receptor found in the cell membrane, that binds pheromone from its neighbouring environment. Pheromone binding activates this heterotrimeric G-protein, which activates signaling through a cascade of MAP kinases, MAP3K, MAP2K, MAPK, to the downstream effector molecule, Fus3. Activated Fus3 phosphorylates the Ste12 transcription factor, which then binds to the pheromone responsive promoter elements, inducing gene transcription and causing morphological transformation into shmoo [3]. Fus3 also activates Far1, which is a cyclin dependent kinase inhibitor in the polarization branch of this pheromone signaling, causing cell cycle arrest in G1 [3, 4]. A small adaptor protein Ste50p interacts with the MAP3K Ste11 by their mutual SAM/SAM domains [5], and without this interaction mating is inefficient, and in some strains reduced about 100-fold [5, 6]. The Ste50p adaptor is known to enhance pheromone signaling, and its overexpression causes supersensitivity to pheromone [6], while a Ste50p null severely reduced *FUS1* activity [6]. Several lines of evidence suggest that Ste50p function is impaired when the c-terminal domain is truncated or contains point mutations; these mutants show reduced transcriptional activation, cell cycle arrest, and shmoo polarization when exposed to pheromone [6, 7]. Additionally, Ste50p localization to the shmoo tip [7] suggests that it might have a direct role in polarization, which has never been explored.

The polarization branch of the pheromone response involves gradient sensing and directional polar growth towards a potential mating partner [8]. However, in the absence of a mating partner, such as when artificially exposed to pheromone, cells polarize at the default polarity site, which is defined as the axial bud site [9–12]. Cell polarity is a highly stratified regulatory process involving many spatially and temporally regulated molecules; among those, Far1 scaffold protein has a fundamental role in determining the site of polarization [13], in which a Far1-Cdc24 complex interacts with Gβγ and recruits Cdc42 to the polarity site at the cell cortex [13]. The GTPase, Cdc42, has a key role in establishing the polarity front or “polarisome” at the apical region with the help of its effectors and regulators [14]. The polarisome includes active Cdc42-GTP, activated by its GEF Cdc24, that binds the scaffold protein Bem1 via a PAK Cla4 [15], to facilitate activation of other Cdc42-GDP molecules and form a polarity complex [16]. Bem1 recruits regulatory proteins, such as an actin-nucleating formin called Bni1, which assembles actin filaments to the site of polarization [17]. Along these actin cables, the V myosin and Myo2p myosin family of molecular motors transport secretary vesicles tethered by the exocyst [18]. Additional mechanisms relating to cell wall expansion and polarization are essential to sustain the mating projection [19]. This feedback is provided by the cell wall integrity (CWI) pathway that uses the stress sensors Wsc1, Wsc2 and Mid2 to activate Rho1, a GTPase [20], that subsequently activates membrane localized glucan syntheses Fks1/2, critically brought to the site of polarization by the secretory vesicles [21]. In many instances, even in the presence of the aforementioned regulatory systems, polarization fails, resulting in a mixture of phenotypes [22–25].

Previously, we found Ste50p localizes at the shmoo tip in our still microscopic studies [7]. Here, we extended our investigation at the single-cell level by time-lapse microscopy to follow the dynamics of Ste50p localization during cell polarization upon pheromone exposure. We show spatiotemporal localization of this protein to the site of polarization and its association with the shmoo during the initiation, elongation and the termination of extension. Our results also show that variation in the cellular level of Ste50p at the G1 phase of the cell cycle influences polarity decisions and causes phenotypic heterogeneity, suggesting, a collaborative action of this protein in the signaling branch and the polarization branch of the pheromone response pathway to effectively control mating.

## Results

### Ste50p polarity patch formation is pheromone concentration-dependent

**Fig 1:**
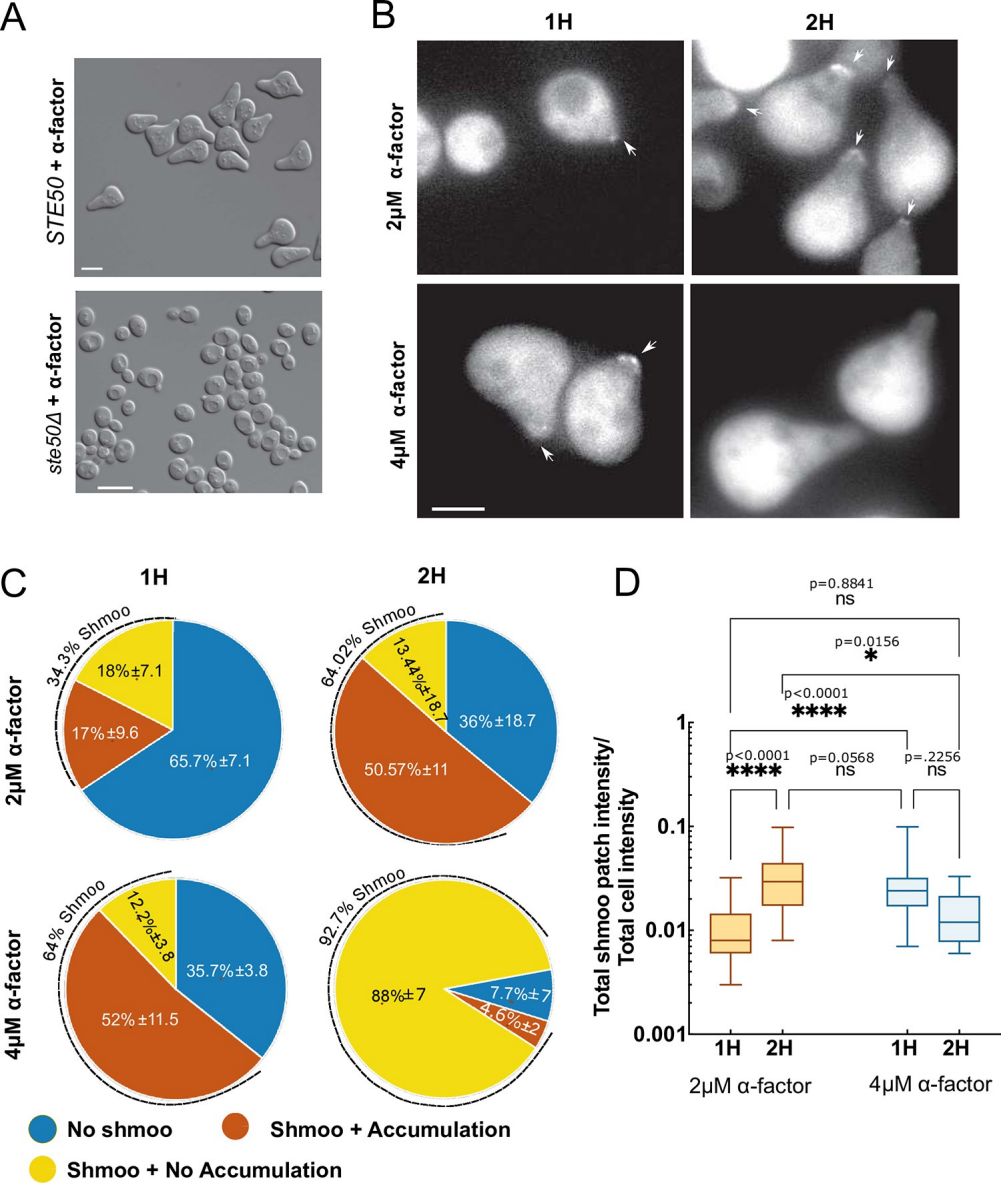
Pheromone concentration-dependent Ste50p shmoo tip localization *ste50Δ* strain, with or without Ste50-GFP on a plasmid, was treated with α-factor and imaged using epifluorescence and DIC microscopy. (A) Response of *STE50* as indicated after 4h treatment with 2μM α-factor. (B) Ste50p patch localization at the shmoo tip at the indicated concentrations of α-factor for the indicated time. (C) Number of cells with localized Ste50p polarity patches at the shmoo tip at the indicated pheromone concentrations and time (n≥100 cells, N = 3). (D) Quantified polarity patches of Ste50p at the shmoo tip with respect to the cytoplasmic amount at indicated pheromone concentrations and time (see text); N = 2: 2μM 1h (mean = 0.01, SD±0.0064; n = 35), 2μM 2h (mean = 0.035, SD±0.0224; n = 75), 4μM 1h (mean = 0.028, SD±0.0163; n = 78), 4μM 2h (mean = 0.015, SD±0.0091; n = 8); ****, p<0.0001; ns = not significant; p>0.05; one-way Anova with Tukey’s multiple comparisons. Bar represents 5μm.

Given that a *ste50Δ* strain fails to polarize, while wild type (WT) cells develop >80% shmoo (Fig 1A) [7], it is reasonable to suggest that Ste50p plays a role in the polarization of yeast cells in response to pheromone. Our previous localization studies have demonstrated that upon 2μM pheromone exposure, a fraction of the cellular Ste50p is recruited to the tip of the growing shmoo [7], while mutants, defective in pheromone signaling and impaired in shmoo formation, failed to localize itself in the scanty shmoo structures [7], suggesting, Ste50p localization at the shmoo tip may be linked to proper polarization.

To re-evaluate and gain insight in this polar localization of the Ste50p along with its differentiation behavior over pheromone concentrations, we used a *ste50Δ*, *bar1Δ* yeast strain (S2 File), and a centromere plasmid containing the GFP-tagged *STE50* gene that is driven by its natural promoter, as in previous studies (S1 Table) [7]. Cells were treated with 2μM or 4μM of pheromone for 1h or 2h then imaged using DIC and fluorescence microscopy to study cellular morphology and the Ste50-GFP localization. Our results clearly showed that pheromone has a dose-dependent effect on the number of the shmoo as well as the size of the Ste50p patches at the shmoo tip (Fig 1B and 1D). We found that after 1h of 2μM pheromone treatment, about 34.3% of cells formed shmoo (SD± 7.09%, ≥200 cells, N = 3), similar to that reported previously [7], and 49% of these shmoo had Ste50p patches at the tip (SD± 9.64%, ≥200 cells, N = 3) (Fig 1C). While a 2h treatment caused a 2-fold increase in shmoo (64% SD± 18.7%, ≥200 cells, N = 3) as well as an increase in the proportion of cells with tip-localized Ste50p patches (79% SD± 11%, 100–200 cells, N = 3) (Fig 1C) [7]. Doubling pheromone concentration (4μM) doubled the amount of shmoo formation at 1h (64.33% SD± 3.79%, 100–200 cells, N = 3), and 81% (SD± 11.5%, 100–200 cells, N = 3) of those shmoo had Ste50p patches at the tip (Fig 1C). Interestingly, although treating cells longer with 4μM pheromone showed an increase in the number of shmoo (92.33% SD± 67.23%, 100–200 cells, N = 3), at 2h there was a drastic decrease in the number of shmoo that had patches at the tip (only 5%; SD± 1.53%, 100–200 cells, N = 3) (Fig 1C and 1D), indicating a transient nature of Ste50p localization at the shmoo tip in response to pheromone.

We quantified the GFP fluorescence in these patches and normalized them as fractions of the total cytoplasmic Ste50p in individual cells at different durations and concentrations of pheromone (Fig 1D). The analysis showed, mean estimated fraction of the Ste50p (total shmoo patch intensity/total cell intensity) patch varied between 1.0% to 3.5% depending on the pheromone concentrations and time (n≥35, N = 2, except for 2h 4μM n = 8) (Fig 1D). Higher pheromone concentration (4μM) encouraged larger patches that appeared sooner and disappeared faster than lower concentration (2μM) (Fig 1B and 1D). Taken together, these results show that the percentage of cells forming shmoo is directly proportional to the pheromone concentration; a graded pheromone response has also been observed previously [26]. Results also demonstrate that the appearance of a discernible polarization patch of the Ste50p at the tip is dependent on pheromone concentration and the length of treatment, with lower pheromone concentration leading to a smaller Ste50p patch that remains for longer period, while higher concentration causes more intense, transient Ste50p tip localization.

### Cortical Ste50p patches are incipient sites for polarization

**Fig 2:**
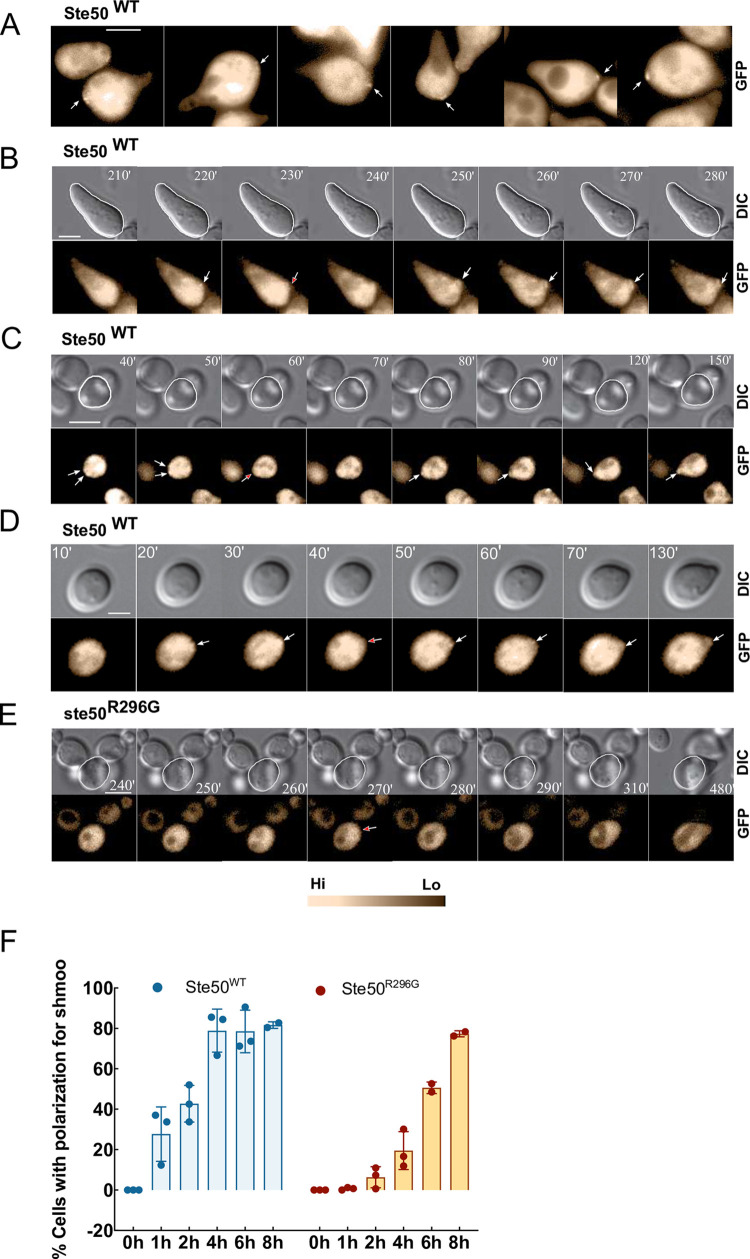
Cortical Ste50p patches are incipient sites for polarization. (A) Ste50-GFP plasmid bearing yeast cells were treated with α-factor and imaged using epifluorescence and DIC still microscopy, showing cortical Ste50p foci (arrow), detectable after ~3h pheromone treatment. (B-D) Single-cell analysis by time-lapse microscopy showing nucleation of the Ste50p on the cell cortex (white arrowheads, S1–S3 Movies) before polarization for a 2^nd^ and 1^st^ shmoo respectively (red arrowheads indicates start of polarization), and mobile patch that stabilizes before 1^st^ shmoo appearance (C, white arrowheads), (E) ste50p mutant that fails to form patch, R296G, has a delayed polarization (red arrowhead indicates start of polarization), time indicated, cell circumference marked in DIC image to show the start of polarization, bar 5μm (S6 Movie). (F) Percentage polarized cells at indicated times for the WT (n>200, N = 3) and the mutant (n>100, N = 3).

In our population level time-course microscopic studies, treating cells with pheromone for more than 2h caused enlargement of the cells, generally after 3h of stimulation, developing a 2^nd^ shmoo (S1A and S1B Fig) [27]. Examination of still images readily detected patches of localized Ste50p on the cell cortex after 1^st^ shmoo formation and ~3h of pheromone exposure [Fig 2A and S1C Fig]. Based on these observations, we hypothesized that Ste50p polarity patches on the cell cortex are incipient sites of shmoo polarization. To test this hypothesis, we carried out time-lapse imaging of yeast cells, expressing Ste50-GFP and exposed to pheromone, at 10 min intervals for 8-12h (see S2 File).

Single-cell studies by time-lapse microscopy showed details of Ste50p translocations within the cell; patches of Ste50p were mobile within the cytoplasm and also presented themselves at the growing shmoo tip where they were found to engage/disengage. By tracking single cells over time, we confirmed that Ste50p formed localized foci on the cell cortex as early as 10 min (S1D Fig) that corresponded to the shmoo initiation sites developing shmoo structures (Fig 2B–2D; S1–S5 Movies). This phenomenon could be detected for both the 1^st^ (Fig 2C and 2D) and the 2^nd^ (Fig 2A and 2B) shmoo, though it was more readily detected in the 2^nd^ shmoo as the patches were larger. Instead of staying engaged at the tip of the growing shmoo, sometimes Ste50p patches transiently disengaged (Fig 2B frame 3 and 4; 2C frame 4). Patches were found to “wander” around the cell cortex, a sign of partner search [28], before stabilizing at a site where cell polarizes shmoo (Fig 2C; S2, S4 and S5 Movies). Cortical patches at the incipient shmoo site could be detected in 64.4% of cells (n = 59) that became polarized. Although single cells differ in cortical patch localization timing after pheromone treatment (between 10-70min, av. = 30.2min, SD = 19.00; n = 50, N = 3), once patch is localized, shmoo polarization tightly followed, generally within a frame or two (av.13.2min, SD = 4.71, n = 50, N = 3).

To determine if cortical Ste50p patches are involved in the initiation of shmoo polarization, the Ste50-RA domain mutant R296G [7] that is severely defective in pheromone signaling (S2 Fig), patch localization at the shmoo tip, and polarization was studied by the time-lapse microscopy after pheromone treatment. We examined the behavior of the GFP-tagged mutant protein R296G, which showed an inability to localize cortical patches at the incipient shmoo site (n = 102) and suffered a significant delay in the initiation of polarization compared to the WT (Fig 2E, S6 Movie). Exposure to pheromone for 1h, 2h, and 4h had initiated polarized growth in only 1.29%, 7.32%, and 19.57% of cells (Fig 2F, n>100, N = 3), compared to 27.71%, 42.55%, and 78.89%, respectively in the WT (Fig 2F, n>200, N = 3). Unexpectedly, the mutant seemed to recover its lack of polarization after prolonged pheromone exposure, and actively engaged in the shmoo formation to attain the WT level (Fig 2E and 2F).

Thus, our single cell time-lapse studies confirmed that Ste50p polarity patches move, present themselves near the presumptive shmoo site where they form foci at the cell cortex, and that the specific arginine residue at position 296 of the Ste50-RA domain is involved in the early shmoo development phase.

### Polarity patches associate with the shmoo until maturation

**Fig 3:**
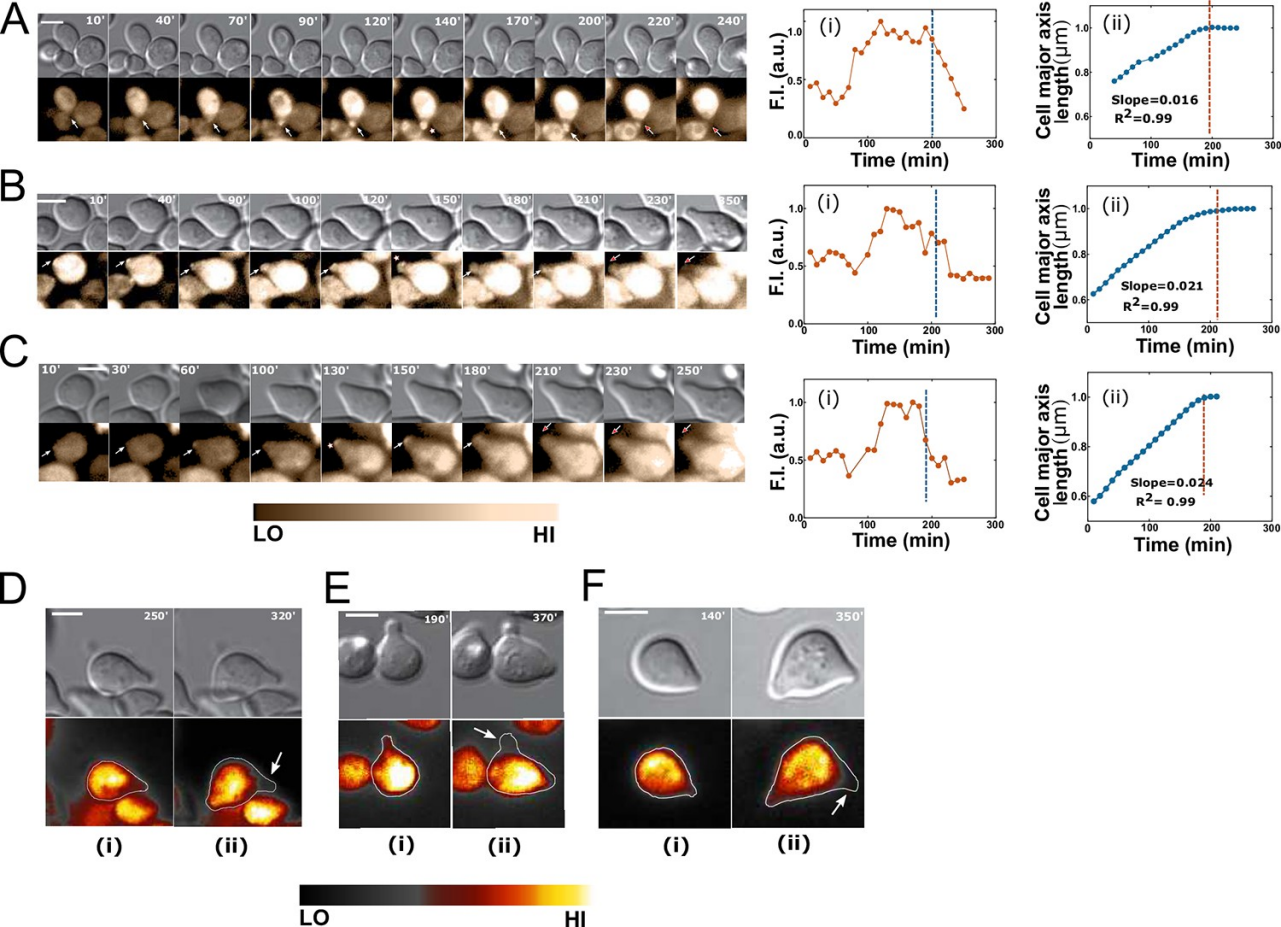
Ste50p localizes to the shmoo tip until shmoo maturation. Ste50p-GFP-expressing cells were treated with 2μM α-factor and imaged using time-lapse microscopy. (A-C) Single cells with indicated intervals in minutes showing Ste50p patches at the shmoo tip (white arrows); the white asterisk (*) indicates signal peak; the red arrow indicates receding of Ste50p from the shmoo (S7–S9 Movies). (Ai-Ci) Quantified GFP fluorescence at the shmoo tip (~0.2–0.3μm^2^ area at the tip; see S2 File) showing a peak Ste50p around 130–200 min after pheromone treatment that starts receding around 200 min (blue broken lines). (Aii-Cii) Correlation between the disappearance of Ste50p from the shmoo and the termination of polarized shmoo growth (orange broken lines indicate start of shmoo growth inhibition; see text); the major cell axis (μm) versus time (min) have been plotted (4 point rolling average); both shmoo Ste50p and growth normalized to the maximum values for each cell; linear regression fit to the linear subset of data (length when shmoo extension started until when it stopped) showing the rate of shmoo growth, slope = 0.016, 0.021 and 0.023, respectively. (Di-Fi) Cytoplasmic Ste50p retracted from the 1^st^ shmoo and relocalized to the 2^nd^ shmoo (pronounced around 270–320 min; white arrow indicates 1^st^ shmoo) (Dii-Fii). Scale bar represents 5μm.

The observation that localization of Ste50p patches at the cell cortex precedes shmoo formation (Fig 2), and that patch disappears from the shmoo tip after prolong pheromone treatment (Fig 1B), raises the question as to whether Ste50p is associated with the shmoo only during the development phase. To answer this question, we used time-lapse microscopy to correlate localization of Ste50p at the shmoo tip with the extension of the shmoo on a cell-by-cell basis. The patches were dynamic; appearing at the shmoo site, remaining associated with the shmoo tip during growth and then disappearing (Fig 3A–3C; S7–S9 Movies), usually discernible patches (between ~0.6-2μm) formed between 100–200 min followed by their gradual disappearance. To find if there is a relationship between the Ste50p at the shmoo tip and the polarized growth, fluorescence intensity at the shmoo tip was quantified and plotted against time; this analysis produced histograms with unimodal peak of Ste50p-GFP intensity in a given shmoo tip (Fig 3Ai–3Di; see S2 File), showing its tip appearance/ disappearance. We then quantified the polarized growth by measuring the long axis of the cell through time (Fig 3Aii–3Cii); this analysis revealed that polarized growth increased linearly with time as long as the Ste50p polarity patches remained at the tip, and plateaued when the polarity patches disappeared (Fig 3Ai–3Ci and 3Aii–3Cii). In our analysis, we used a regression fit to the linear subset of data to find the slope that may be interpreted as the rate of shmoo elongation, which shows polarization rate is different between cells (Fig 3Ai–3Ci and 3Aii–3Cii), possibly contributing to phenotypic heterogeneity.

Notably, at maturation (time when shmoo stopped extending), not only polarity patches at the shmoo tip disappeared, but also cytoplasmic Ste50p retracted from the shmoo approximately after 210–230 min (Fig 3A–3C). This was strikingly evident in the case of a 2^nd^ shmoo formation, as Ste50p retracted itself from the 1^st^ shmoo and then redirected to the 2^nd^ shmoo (Figs 3D–3F and S3), leaving the 1^st^ shmoo devoid of Ste50p. In addition to this phenomenon being present during the 2^nd^ shmoo formation, we could also detect this phenomenon in cells bearing only one shmoo after its maturation (Fig 3A–3C). Our earlier observations involving cells, stimulated for 2h at 4μM pheromone, showed patch association with the shmoo in only 5% of cells (Fig 1B and 1D), reinforcing our hypothesis that at higher pheromone concentration shmoo matures earlier and thus Ste50p departs sooner. Therefore, both the initiation and the termination of polarization in cells correlates with the presence and absence of Ste50p respectively, suggesting possible involvement of Ste50p in these events. Our results demonstrate that Ste50p is present in the shmoo only during shmoo development until maturation and its further presence is not required for shmoo retention.

### Increased Ste50p expression in response to pheromone synchronizes with polarization

**Fig 4:**
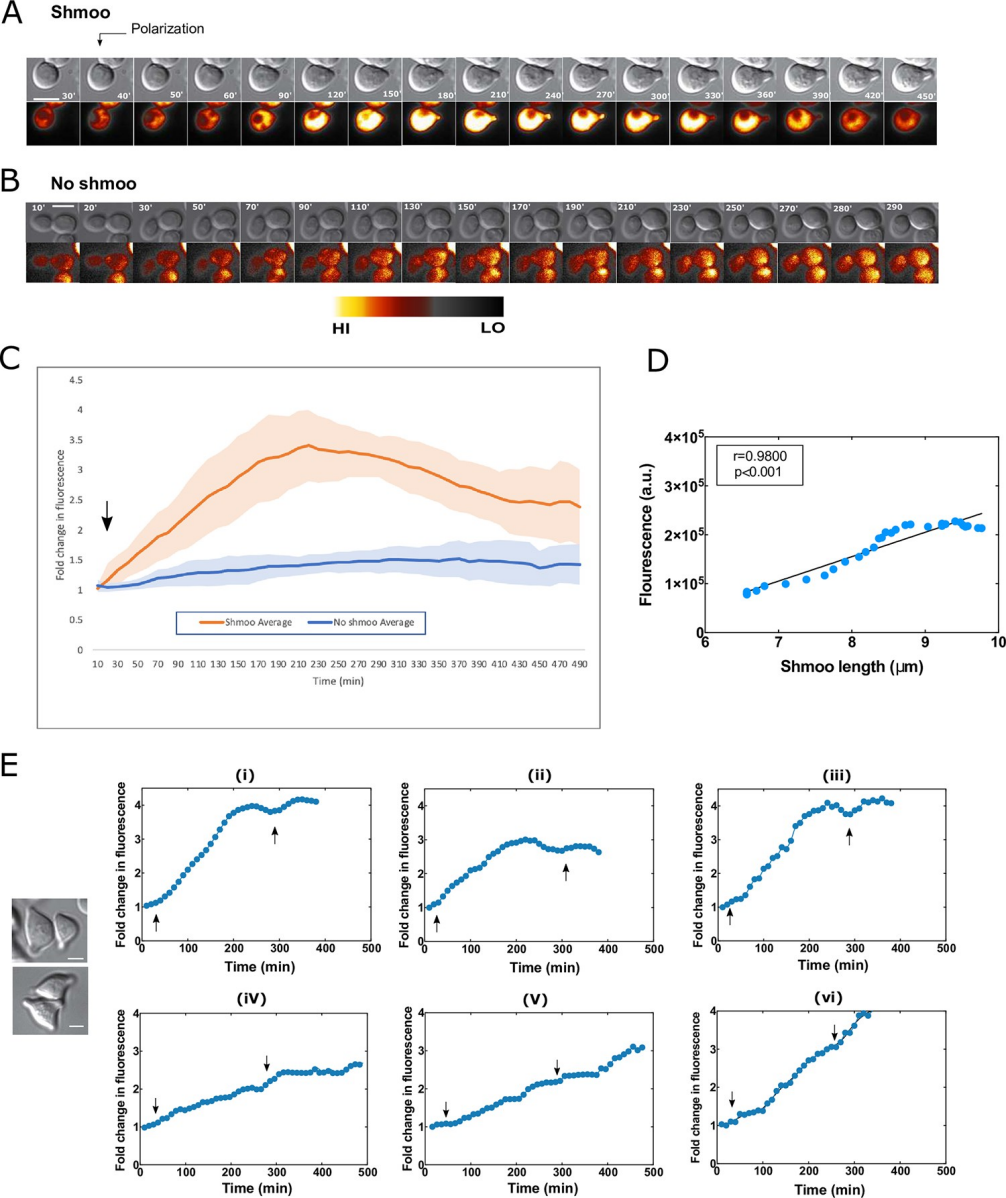
Increased Ste50p expression synchronizes with shmoo polarization. Yeast cells expressing Ste50p-GFP were treated with 2μM α-factor and followed by time-lapse microscopy for at least 8hrs. (A) Shmoo-forming cells show an increase in Ste50p expression during polarization (arrow as indicated) (S10–S12 Movies), in contrast to cells that do not form shmoo (S13–S15 Movies) (B); time as indicated, bar 5μm. (C) Quantified cellular GFP fold changes in single cells across time, showing an unimodal expression peak at ~150–270 min for single shmoo forming cells (orange, C, arrow indicates start of polarization); n = 10; N = 3, and no significant changes in no shmoo forming cells (blue, C); n-10; N = 3.; shadings are standard deviations. (D) Correlation between Ste50p expression and shmoo growth of cell in A; Pearson r = 0.9800, p<0.001. (E) Bimodal and linear increase of Ste50p expression in the cases of multiple shmoo (see text, arrows indicating the onset of 1^st^ shmoo and 2^nd^ shmoo).

During our analysis, we consistently found some cells to undergo shmoo polarization, while others were cell cycle arrested (CCA) with no polarization in response to pheromone. To investigate whether the decision for the phenotypic transformation was associated with changes in the Ste50p expression level, we qualitatively examined GFP fluorescence of single cells across time. We analysed both CCA cells and post-Start dividing cells that were programmed to complete the cell cycle [29] and arrive at G1 phase to polarize. Examination of single cells discovered a striking rise in the Ste50p level at the onset of polarization in the shmoo forming cells in response to pheromone (Fig 4A; S4A and S4D Fig; S10–S12 Movies). In contrast, cells that remained refractory to pheromone clearly showed no significant increase in the Ste50p level across time (Fig 4B; S13–S15 Movies). To have a quantitative assessment of this expression increase, we measured fluorescence in single cells for 8hrs from time-lapse movies. In this analysis, we included cells from only two groups that formed a single shmoo: (i) cell cycle arrested single cells that formed shmoo, (ii) dividing cells that arrived at G1 to shmoo. To keep consistency and a baseline for comparison, our analysis also incorporated few time-lapse frames before cell separation in dividing cells. To contrast, we also measured GFP levels in the undifferentiated cells across time. Fluorescence quantification clearly showed that when cells became pheromone responsive and initiated polarization, they displayed a 3.42-fold (SD±0.075) increase in the Ste50p expression levels that was concurrent with the shmoo extension (Fig 4C, S4B and S4E Fig) and exhibited a unimodal peak around 170–290 min for a single shmoo forming cell in a histogram (Fig 4C, orange). In contrast, the undifferentiated cells showed unremarkable Ste50p level changes across time (Fig 4C, blue). This rise in the Ste50p level positively correlated with the shmoo extension until shmoo maturation (Fig 4D corresponding to the cell in 4A; S4C and S4F Fig), further reinforcing our results for shmoo maturation in the previous section (Fig 3), and extending it to the overall loss of Ste50p from the shmoo. On the other hand, cells that are committed to form a 2^nd^ shmoo, often showed a distinct bimodal histogram, with peaks coinciding with the formation of 1^st^ and 2^nd^ shmoo around ~150–270 min and 300–400 min respectively (Fig 4Ei–4Eiii). Some multi shmoo forming cells showed a sustained increase in levels of Ste50p across time (Fig 4Eiv–4Evi). Among the 35 single cells that were analyzed for the Ste50p expression across time (up to 8–12 hours), 30 adhered to this phenomenon and displayed a temporally regulated increase (“burst”) of Ste50p during shmoo polarization. Taken together, these results strongly suggest that Ste50p is a pheromone responsive gene and increased Ste50p level is associated with the morphological transformation required for shmoo polarization.

### High levels of Ste50p at G1 correlate with ensured polarization

**Fig 5:**
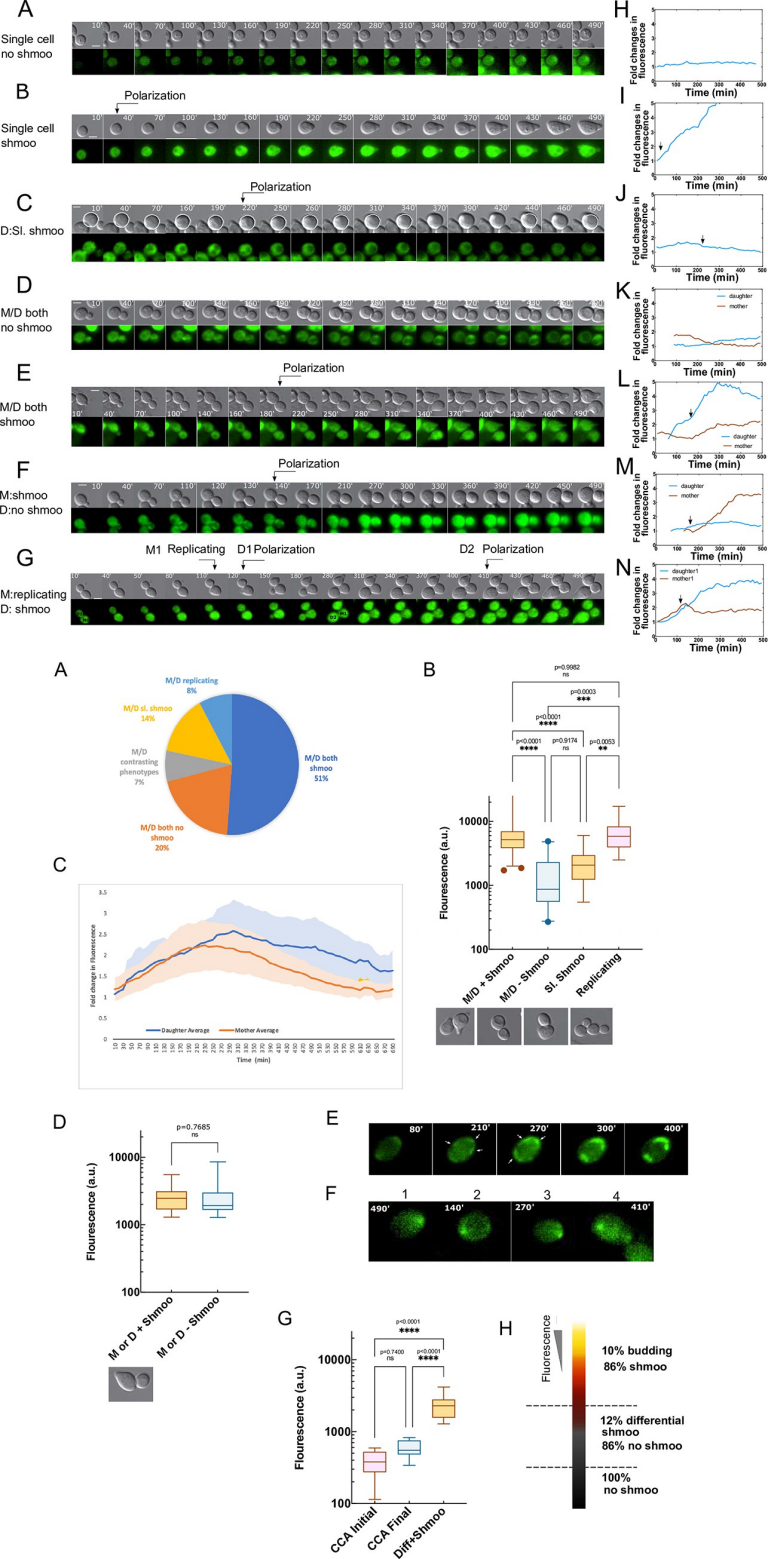
Phenotypes of yeast cells after pheromone treatment. Part I. Yeast cells were exposed to 2μM α-factor and phenotypic changes were examined by time-lapse microscopy for eight hours. (A-G) Representative DIC and fluorescence images of each phenotypic category found, as indicated, polarization time indicated by arrow, M = mother and D = daughter, sl. = slight, cell circumference in white to show directional growth or polarization (see text), time as indicated, bar 5μm, (S16–S22 Movies). (H-N) Quantified fold induction of the Ste50p fluorescence over time for the corresponding cells in (A-G). Arrow indicates beginning of detectable polarized growth. Part II. Polarization correlates with high levels of Ste50p at G1. Yeast cells were treated with 2μM α-factor and followed by time-lapse microscopy for at least 8hrs. (A) Percentage of different phenotypes observed (n = 211 cells, N = 3). (B) Fluorescence quantified at G1 immediately after M/D separation for the indicated phenotypic groups, showing significant difference in Ste50p level among the groups; higher level correlates with polarization in the M/D shmoo group (see text), N = 2: M/D shmoo (mean = 6995, SD±5824; n = 108), M/D no shmoo (mean = 1440, SD±1142; n = 42), slight shmoo (mean = 2109, SD±1202; n = 29), replicating (mean = 6791, SD±4070; n = 17); one-way Anova followed by Tukey’s multiple comparisons. Lower panel: DIC images of the corresponding phenotypes, bar 5μm. (C) Quantified fold changes in fluorescence in mother and daughter from time-lapse movies; N = 2, n = 14; shadings are error bars for standard deviations and solid lines are means as indicated. (D) Ste50p expression at G1 for contrasting phenotypic M/D as indicated; N = 2, n = 15 in each case; unpaired Student’s t-test, ns, p = 0.7685; shmoo (mean intensity = 2759, SD±1262); no shmoo (mean intensity = 2592, SD±1763). Lower panel: DIC images of the phenotypes. (E) Time-lapse frames as indicated showing mobile Ste50p-GFP foci on the cell perimeter and their stabilization, arrows pointing at patches (S23 Movie). (F) Formation of Ste50p foci in CCA cells after prolonged pheromone treatment, but unable to polarize, time indicated (cells 1–4). Bar represents 5μm. (G) Quantified GFP for CCA cells at 0hr (initial) and 8hrs (final) of pheromone exposure, forming foci but unable to polarize; n = 9, in comparison to M or D + shmoo in (D), one-way Anova, followed by Tukey’s multiple comparison test, mean intensities, initial = 388, SD±155; final = 586, SD±164; M or D + shmoo = 2759, SD±1262. Y-axis in log scale to expand the values. (H) Representative heat map of Ste50p fluorescence versus percentage of shmoo forming cells.

Our time-lapse studies revealed a mixture of different phenotypes in the cell population after 4h pheromone treatment. The different phenotypes were: single cells without shmoo; single cells with shmoo; vegetatively replicating cells; mother/daughter (M/D) both with shmoo; M/D both with no shmoo; M or D shmoo; cells with only slight shmoo (incomplete polarization with decrease in cell sphericity and a small pointed front; complete shmoo when polarization is close to a bowling pin like structure) (Fig 5 Part I A-Part I G, S16–S22 Movies). The cellular GFP fluorescence was quantified in these phenotypes and plotted over 4h (Fig 5 Part I H-Part I N), which show different profiles of the Ste50p expression among each phenotypic category (Fig 5 Part I H-Part I N). These observations raised the question, does phenotypic heterogeneity in response to pheromone depends on the Ste50p level at G1 before differentiation begins? Furthermore, is there a minimal level of Ste50p required to differentiate? To answer to these questions, we focused on M/D pairs, since they could serve as a system to study the initial GFP levels at G1 immediately after cell separation for Ste50p expression analysis and phenotypic heterogeneity. To perform this analysis, we rejected dead cells, identifying them by their morphology or as cells having no movements inside (visible in DIC), and considered M/D pairs that moved away from each other after cell separation. Within the 211 M/D dividing pairs analyzed, 108 M/D both had shmoo, 42 M/D both had no shmoo, 15 M/D had contrasting phenotypes, 29 had slight shmoo, and 17 were replicating (Fig 5 Part II A). Between the M/D, in about 7% of the time, contrasting phenotypes of shmoo or no shmoo were observed. To find if there is any relationship between the level of Ste50p at G1 prior to polarization and the phenotypic fate, we analyzed the different phenotypic pools described above and quantified their cellular GFP at G1 upon M/D separation (Fig 5 Part II B). Our results show that there is a significant difference in the Ste50p levels between the shmoo or the no shmoo M/D; cells with higher Ste50p levels (shmoo, mean intensity = 6995, SD±5824) were committed to polarize, and in rare cases, high Ste50p levels (mean intensity = 6791, SD±4070) favored replication. On the other hand, cells with low Ste50p levels either formed slight shmoo (mean intensity = 2109, SD±1202) or no shmoo (mean intensity = 1440, SD±1142) (Fig 5 Part II B). This demonstrates that the level of Ste50p at the initial G1 correlates with the different phenotypic outcomes, non-dividing cells with high Ste50p levels commit to polarization, and cells with low Ste50p levels remain mostly undifferentiated.

To further dissect and find if there is a difference in the Ste50p expression between the mother and daughter across time when both are forming shmoo and at a similar Ste50p expression level at G1, we quantified fluorescence in them from time-lapse movies over extended period of time. The analysis revealed that despite almost identical initial increments of the Ste50p fold induction between M and D, daughters were delayed in reaching peak intensity, however sustained increased fold induction of GFP after reaching the peak level for longer period than the mothers (mother n = 14, daughter n = 14) (Fig 5 Part II C). This delay in reaching peak intensity for the daughters may indicate time for their maturation, while their sustained increased level in the later phase could indicate cellular vigor due to the absence of replicative aging [30], yet maintaining a correlated expression pattern in successive generations.

The M/D contrasting phenotypic group (either mother or daughter forms shmoo) showed low levels of Ste50p at G1 for both the shmoo forming (mean intensity = 2268, SD±839) and no shmoo forming cells (mean intensity = 2402, SD±723) (Fig 5 Part II C), suggesting that at this Ste50p level, cells have an option for choosing cell fates; cells could polarize or stay undifferentiated, a decision made with no particular preferences between the M/D. While higher Ste50p level correlates with obligatory polarization. In the time series, some of the single CCA cells that are not differentiated into shmoo, showed very low levels of Ste50p (mean initial intensity = 388, SD±155; mean final intensity = 586, SD±164). These cells have been very informative to follow movements of Ste50p polarity patches across time (Fig 5 Part II D and Part II E), and showed initial patch surveillance around the perimeter of the cells that subsequently stabilized to form focused Ste50p patches (Fig 5 Part II D, S23 and S24 Movies; also, S2 and S4 Movies). Multiple Ste50p clusters initially existed in some cells (S23 Movie) that coalesced to form a focused front, however, cells finally failed to polarize even after 8h of 2μM pheromone exposure, identifying them as the CCA undifferentiated cells. The fluorescence intensities in these cells were considerably lower (Fig 5 Part II G) than the fluorescence where cells had a slim possibility to shmoo (Fig 5 Part II D), demonstrating that a minimal level of Ste50p correlates to the symmetry breaking for polarization (Fig 5H). Increasing the level of Ste50p is associated with a tendency in cells to polarize, and finally at higher levels of Ste50p, cells are found to be mostly polarized (86% shmoo and 10% budding) (Fig 5 Part II H).

## Discussion

Pheromone exposure causes yeast cells to polarize into mating projections for conjugating with a mating partner. To date, major players in yeast polarization have been elucidated, such as Cdc42, Bem1, PAK, and Cdc24; these form polarity complexes in the cytoplasm, move freely [15, 16, 31–33], and also congregate at the membrane [15]. Our previous study elucidated that WT Ste50p patches associate with the growing shmoo tip, while Ste50p mutants defective in pheromone signaling were impaired in patch association [7], suggesting patch localization is critical for proper polarization of yeast. The present work strongly supports this hypothesis. Using single-cell fluorescent microscopic studies of the spatiotemporal localization and expression of the Ste50p during the polarization of yeast cells in response to mating pheromone, we systematically show that this protein is associated with the initiation, elongation, and the termination of the polarized shmoo structure. Polarization is synchronized with the Ste50p expression burst in response to pheromone, and the differential levels of the Ste50p among individual cells is significantly correlated with cells’ ability to polarize, hence, suggesting a possible role in the co-existence of mixed phenotypes in the cell population.

Our previous work demonstrated the existence of the Ste50p patches at the shmoo tip of polarizing yeast cells [7]; in the current study we first characterized the appearance and disappearance of these patches in response to increasing pheromone concentrations. This analysis revealed that higher concentration induced rapid polarization (number of shmoo doubled at 1h compared to the lower pheromone treatment), and localization of larger patches more transiently at the shmoo tip than lower concentration (Fig 1C and 1D). These results show that Ste50p patches are associated with the shmoo during its development phase, higher pheromone maximizes shmoo formation earlier, hence, patches disappear sooner, indicating that Ste50p may be involved in the shmoo structure formation of yeast cells.

Subsequently, we carried out time-lapse imaging of pheromone-treated yeast cells that revealed cortical nucleation of the Ste50p at the presumptive shmoo site as early as 10 min after pheromone treatment prior to polarization. Ste50p patches were found to engage/disengage (Fig 2) at the cortical site, an “oscillatory” behavior also found for Bem1p, which has been linked to the negative feedback mechanism for vegetative polarization [34], suggesting a similar process for Ste50p in the shmoo polarization. During the initial stages of polarity establishment, sometimes patches were found to move along the perimeter of the cell, finally stabilizing at a site where the symmetry was broken for polarization (Fig 2C, S2–S4 Movies); this behaviour is typical of polarity proteins in *S*. *cerevisiae* [26, 35], which has been linked to the partner search process [28]. The patch wandering and movements could be driven by the V myosin vesicle transport along the actin cables to the polar site [21, 36–39]. Actin patches are formed at the cortical membrane zones together with the regulatory proteins that bind actin [40]. Studies with Cdc42p in polarity establishment during budding showed evidence that Cdc42p patch formation at the presumptive bud site is independent of the localization or integrity of the actin cytoskeleton [14]. However, whether the Ste50p localization at the presumptive shmoo site is actin dependent/independent needs to be further investigated. Besides actin, interactions with other polarity establishment proteins such as Cdc42p, Bem1, Ste5 and Far1 [41] may provide a control mechanism for the Ste50p subcellular localization. Indeed, Cdc42p is known to bind to Ste50p [42, 43], though bioinformatic predictions showed that mutation of Ste50p at sites other than Cdc42p binding sites caused loss of polarization [7], existence of other spatiotemporal interactive sites between these two proteins, or through a mediator protein that are critical for polarization is highly possible.

Under normal circumstances, the pheromone signal amplification by Ste50p is critical for pathway function and polarization, since a *ste50Δ* or *ste50* mutants grossly attenuate *FUS1* promoter response and shmoo formation (Fig 1A) [6, 7]. By time-lapse movies of yeast cells for extended period of time, here we show that a ste50p mutant (R296G) defective in *FUS1* promoter response suffer a considerable delay in the initiation of shmoo with respect to the wild type (Fig 2F). Additionally, overexpressed Ste11p in a *ste50Δ* strain showed a delay in polarization [44]. In line with these results, here we reveal that patches of Ste50p localize at the shmoo initiation site prior to the onset of polarization, showing the requirement of Ste50p in the early shmoo development phase. A possible hypothesis arising from these findings is that by interacting with other polarity proteins, Ste50p facilitates the formation of functional polarisome at the membrane to accelerate the polarization process.

Along with the discovery of the Ste50p patches at the initiation site, we also found a timing for the Ste50p patch appearance/disappearance during the shmoo growth that closely correlated with the shmoo initiation and maturation. Ste50p patches appeared at the presumptive shmoo site, and persisted with the growing shmoo until the cessation of shmoo extension (Fig 3A–3C); similar behaviour has been observed for actin in the vegetative bud polarization patch during bud maturation [45]. Interestingly, in multiple shmoo forming cells, Ste50p retracts from the 1^st^ shmoo and redirects into the 2^nd^ shmoo during the development of the 2^nd^ shmoo structure (Fig 3D–3F), suggesting that Ste50p is involved during the development of the shmoo and not required for its maintenance.

We found Ste50p to be upregulated in G1 arrested cells when pheromone responsive genes are actively expressed (Fig 4A and 4C). A rise in the Ste50p expression coincided with the emergence of the shmoo structure (Fig 4A and 4C) and this temporally increased Ste50p expression (burst) could be positively correlated with the shmoo extension (Fig 4D). In contrast, the no shmoo forming CCA cells failed to produce a Ste50p expression burst, further confirming a correlation between the increase in Ste50p expression and polarization. This phenomenon was consistent and tightly regulated, an emergence of a shmoo could be immediately anticipated during an increase in the Ste50p level, and in cases of sequential multiple shmoo formation, sustained increasing levels of the Ste50p was critically linked with the shmoo emergences (Fig 4E). This induction of the Ste50p may cause supersensitivity to pheromone [6] and an upregulation of *FUS3*, previously found to be positively upregulated by feedback mechanism [46]. Thus, *STE50* is similar to many genes that are linked in the G-protein coupled pathway being responsive to pheromone [47]. The magnitude of these expression bursts is variable among cells and are possibly dependent on the *STE50* promoter activation/deactivation in the individual single cells [48]. Pheromone response mediated through promoter activation requires binding of Ste12p to the upstream pheromone response elements (PRE). Organizationally, PREs are multiple, and binds a multimerized activated Ste12p, this is specifically true for pheromone responsive genes that are common to both the haploid cells, *MAT*a and *MAT*α [49]. Although, global expression analysis showed induction of more than 200 genes after pheromone treatment [46, 50], among them, many strongly induced gene promoters lack the predicted number of consensus sequences of multiple PREs for the Ste12p, yet significant others, including ASG7, FIG2, FIG3 completely lack PREs [51]. Whether *STE50* possesses upstream PRE consensus sequences to bind the Ste12p has not been determined. This will need to be further empirically established.

The phenotypic heterogeneity observed among single-cells at G1 when exposed to pheromone, correlated well with the cellular Ste50p levels (Fig 5C). We show that a high Ste50p level is significantly correlated to polarization, both budding and shmoo, selecting between these two may require involvement of additional factors. However, when both mother and daughter formed shmoo, an initial surge in the Ste50p expression was followed by a sustained increased level of Ste50p in the shmoo forming daughters, and a correlated reduced level in the shmoo forming mothers (Fig 5 Part II B), indicating a possible link between the Ste50p expression pattern and the MAPK activity pattern previously found between mother and daughter [52]. Our analysis of the Ste50p expression in the CCA cells lacking shmoo showed very low level of Ste50p, yet these cells showed Ste50p patch clusters in response to pheromone, supporting previous findings for actin patch clusters [36], and merging of clusters [9] to finally form a polarity front after prolonged pheromone treatment (Fig 5 Part II D, S23 and S24 Movies). We found that the time to form a stable Ste50p patch showed considerable cell-cell variability when Ste50p levels were low, and were in hours rather than in minutes as found for the Bem1p in vegetative polarization [34], However, even with distinct Ste50p foci on the cell cortex at low cytosolic Ste50p levels, cells were unable to break the symmetry and polarize, indicating that a minimal threshold crossing level of Ste50p may be required (Fig 5 Part II G).

The relationship between expression and phenotypic changes has been extensively studied by many in prokaryotic and eukaryotic systems that found noise in expression to be intrinsic or extrinsic, intrinsic noise can arise due to the biochemical processes of transcription or translation [22–25, 53–57]. One of the early views constitute bursty expression of the competitive effectors that may be limiting, causing to partition them between the cells by chance, which compels cells to switch into alternative pathways with phenotypic consequences [58]. Whereas, extrinsic noise includes fluctuation of the components among the transcriptional regulatory network, chromatin remodeling, or segregation of proteins upon cell division [24, 25, 53]. One of the most immediate sources of extrinsic noise in our study is the position of the cells in the cell-cycle allowing heterogenicity. However, since post-Start dividing cells at G1 were analyzed for Ste50p expression and phenotypes, this extrinsic noise was eliminated. Another extrinsic noise could be due to the plasmid-based system. However, we tried to minimize this by using a low copy *CEN* plasmid. Our results here demonstrate that Ste50p expression levels cause a dose-dependent phenotypic variability in yeast cells. Results also show the existence of inherent cell-cell variability in the Ste50p expression level that can be correlated with specific phenotypes, for example, the extremely low levels of Ste50p found in CCA cells with capability to form patches but no shmoo (Fig 5 Part I A, Part II E and Part II F), contributed to the heterogeneity. In extreme cases between the M/D pair, differential all-or-null shmoo formation existed that correlated with striking differences between them in Ste50p fold inductions across time (Fig 5 Part I F and Part I M). The cell fate here could be due to the intrinsic genetic factors or the above-mentioned partitioning of competitive effectors between M/D. Noisy Ste50p levels could be promoter mediated noise that is dependent on transcription factor binding [24]. Fus3p is a downstream component in the pheromone signaling pathway whose activation suffers if upstream Ste50p is lacking, propagating to a decreased Ste12p induction, lack of promoter activation, and reduced cellular level of polarity effector proteins, and as discussed above, expression of Ste50p could be modulated by it. The Ste50p expression heterogeneity among cells is not a snapshot of stochastic gene expression, rather a continuous display of differences in expressions among cells that could be cell specific. This allows to filter out signals of insufficient magnitude or noise, and respond when threshold level is reached.

## Conclusion

In summary, our findings reveal early Ste50p patch recruitment at the site of the mating polarization, indicating the likely involvement of Ste50p in the polarisome, playing a critical role in the timely initiation of polarization. Single-cell data were pivotal in understanding the dose-response of Ste50p expressions that correlated to unique cell-fate progression and phenotypic variability. Our results show how expression of an upstream component in the pheromone signaling pathway may be involved in phenotypic decision that may weed out fitness defective cells in the case of any potential mating event.

## Supporting information

S1 File S1 Macro used for cell fluorescence quantification. (PDF)

S2 File (DOCX)

S1 Table List of plasmids used in this study. (DOCX)

S1 Fig Prolong pheromone treatment causes a 2^nd^ shmoo tip Ste50p. (DOCX)

S2 Fig Transcriptional activation of Ste50p mutant R296G. (DOCX)

S3 Fig Ste50p retracts from the shmoo after shmoo maturation. (DOCX)

S4 Fig Increased Ste50p expression correlates to the polarized extension. (DOCX)

S1 Movie Ste50p patch at the incipient site of polarization. (AVI)

S2 Movie Ste50p patches at the incipient site of polarization. (AVI)

S3 Movie Ste50p patch at the incipient site of polarization. (MOV)

S4 Movie Ste50p patch movement at the presumptive shmoo site. (AVI)

S5 Movie Ste50p patch movement at the presumptive shmoo site. (AVI)

S6 Movie Ste50p mutant R296G polarization. (MOV)

S7 Movie Ste50p associates with the shmoo until shmoo maturation. (AVI)

S8 Movie Ste50p associates with the shmoo until shmoo maturation. (AVI)

S9 Movie Ste50p associates with the shmoo until shmoo maturation. (AVI)

S10 Movie Increased Ste50p level in the alfa factor treated shmoo forming cells. (AVI)

S11 Movie Increased Ste50p level in the alfa factor treated shmoo forming cells. (AVI)

S12 Movie Increased Ste50p level in the alfa factor treated shmoo forming cells. (AVI)

S13 Movie Ste50p expression in no shmoo forming cells. (AVI)

S14 Movie Ste50p expression in no shmoo forming cells. (AVI)

S15 Movie Ste50p expression in no shmoo forming cells. (AVI)

S16 Movie Single cell forming no shmoo. (MOV)

S17 Movie Single cell forming shmoo. (MOV)

S18 Movie Cell slight shmoo. (MOV)

S19 Movie M/D both forming no shmoo. (MOV)

S20 Movie M/D both forming shmoo. (MOV)

S21 Movie M/D forming shmoo/no shmoo. (MOV)

S22 Movie M/D with shmoo/replication. (MOV)

S23 Movie Mobile Ste50p patches on the cell perimeter. (AVI)

S24 Movie Patch movement. (MOV)
