# Supplementary material for: Polarization and cell-fate decision facilitated by the adaptor Ste50p in Saccharomyces cerevisiae
Source: PLoS One. 2022 Dec 20;17(12):e0278614. doi: 10.1371/journal.pone.0278614 (PMC9767377; doi:10.1371/journal.pone.0278614)
Supplement: S2 File — (DOCX) [file pone.0278614.s002.docx]

## Materials and Methods

**Yeast strain, plasmids and transformations**

The yeast strain used in this study was: YCW1886 (*MAT***a** *ste50∆*::*Kan^R^* *ssk1∆*::*Nat^R^ sst1∆*::*hisG* *FUS1-LacZ*::*LEU2* *his3 leu2 ura3 trp1 ade2*), which is *bar1Δ, ste50Δ and ssk1Δ*. The plasmids used in this study are listed in S1 Table. All yeast transformations were carried out using the lithium acetate method (Chen *et al*., 1992). Standard manipulations of yeast strains, culture conditions and media were as described (Dunham *et* *al*., 2015). *E. coli* strain DH10B: F^–^ *mcr*A Δ(*mrr*-*hsd*RMS-*mcr*BC)φ80*lac*ZΔM15Δ*lac*X74 *rec*A1 *end*A1*ara*D-139Δ(*araleu*)7697 *gal*U *gal*K λ^–^ *rps*L(Str^R^) *nup*G (Invitrogen) was used for plasmid maintenance.

**Time-course microscopy of live yeast cells**

Yeast cells bearing Ste50-GFP on a centomeric plasmid were grown to saturation on synthetic defined media without histidine, and then diluted to 1:1000 in fresh media for overnight growth to get exponential cultures the next day. Cells were then treated with 2μM α-factor and samples collected at 1 hour intervals up to 4 hours and prepared for imaging. Still images were captured using a Leica DM6000 equipped with DIC optics, a mercury lamp, and a FITC filter cube (480/20nm ex - 510/20nm em) using Volocity software (Perkin-Elmer, MA, USA), a Hamamatsu Orca ER camera and a 100x PLAN FLUO lens (NA 1.3). The DIC (Differential Interference Contrast) and the GFP images were viewed and analyzed and processed by ImageJ software (v. 1.37; National Institutes of Health).

**Time-lapse microscopy of live cells**

For time-lapse experiments, yeast strains (YCW1886) bearing plasmids were cultured in SD-His media to saturation then diluted into fresh media to obtain exponential culture next morning. One ml of overnight culture was concentrated and cells were loaded onto a multiwell glass-bottom dish (Mattek, MA, USA) pre-coated with concanavalin A (1mg/ml) (Sigma-Aldrich, Oakville, Canada). After cell attachment, cells were covered with 1% agarose (Sigma-Aldrich, Oakville, Canada) at 30^o^C containing 2μM α-factor in thin layer and supplemented on top with 1 ml of SD-His media. Just before viewing, 1 ml of SD-His media with α-factor was added to a final concentration of 2μM. Images were captured on a Nikon Ti microscope equipped with a TIRF arm, DIC optics, a GFP filter cube (480/40nm ex - 520/75nm em), 488nm laser (50mW), a Photometrics Evolve 512 EMCCD camera and a 100x APO TIRF objective lens (NA 1.49). The TIRF arm was adjusted to generate a highly inclined laminated optical sheet (Tokunaga M, *et al*., 2008), and images were captured at multiple XY positions every 10 minutes for 8-12 hours; imaging was performed at room temperature.

**Image analysis**

The ratio of intensity between the shmoo patch and the whole cell was determined by measuring the total intensity of each compartment using FIJI. Briefly, the boundaries of the cell were determined using an automatic thresholding method, verified by the investigator, while the boundary of the shmoo patch was selected by the investigator using the ellipse tool; total intensities were measured for each area and a ratio was calculated (S1 File). Multiple cells were measured per field of view, and all cells were imaged using the same exposure times and fluorescent lamp intensities. Macro used for this analysis is attached in the supplimentary data. Cells that showed fluorescence were counted and quantified, dead cells were rejected and shmoo head out of focus were not counted.

Shmoo tip Ste50p analysis for maturation: ~0.2-0.3μm^2^ area was selected by hand with Imagej by the ellipse tool at the shmoo tip. Then the mean intensity of this area was measured, and corrected for background intensity for a similar size area. Corrected mean intensity was normalized by dividing with the minimum intensity found by the analysis.

The major cell axis was calculated by using the ellipse tool in ImageJ to encompass the whole cell of interest, then measuring the long axis of the ellipse; this analysis was repeated across multiple timepoints.

Ste50p expression was analyzed by using the free hand selection tool in imageJ to encompass the whole cell of interest, then a similar area was measured in the cell background for correction. This analysis was repeated across multiple timepoints. The corrected mean intensity was then normalized against the lowest mean to get fold induction. Cells that were budding were chosen to have a reference point at the cell separation. Frame every 10 min.
