# Supplementary material for: Polarization and cell-fate decision facilitated by the adaptor Ste50p in Saccharomyces cerevisiae
Source: PLoS One. 2022 Dec 20;17(12):e0278614. doi: 10.1371/journal.pone.0278614 (PMC9767377; doi:10.1371/journal.pone.0278614)
Supplement: S1 Table — (DOCX) [file pone.0278614.s003.docx]

**TABLE S1:** List of plasmids used in this study

| Plasmids | Descriptions | Sources |
| --- | --- | --- |
| pCW267(WT) | pRS316-*STE50*^wt^::URA3/AmpR | Wu *et al*., 1999 |
| pRS313-GFP | pRS313-*STE50*-GFP::*HIS3*/AmpR | Slaughter *et al*., 2008 |
| pRS313-GFP | pRS313-*STE50^R296G^*-GFP::*HIS3*/AmpR | Sharmeen *et al*., 2019 |
