# Supplementary material for: Polarization and cell-fate decision facilitated by the adaptor Ste50p in Saccharomyces cerevisiae
Source: PLoS One. 2022 Dec 20;17(12):e0278614. doi: 10.1371/journal.pone.0278614 (PMC9767377; doi:10.1371/journal.pone.0278614)
Supplement: S1 Fig — (DOCX) [file pone.0278614.s004.docx]

**S1 Figure.**

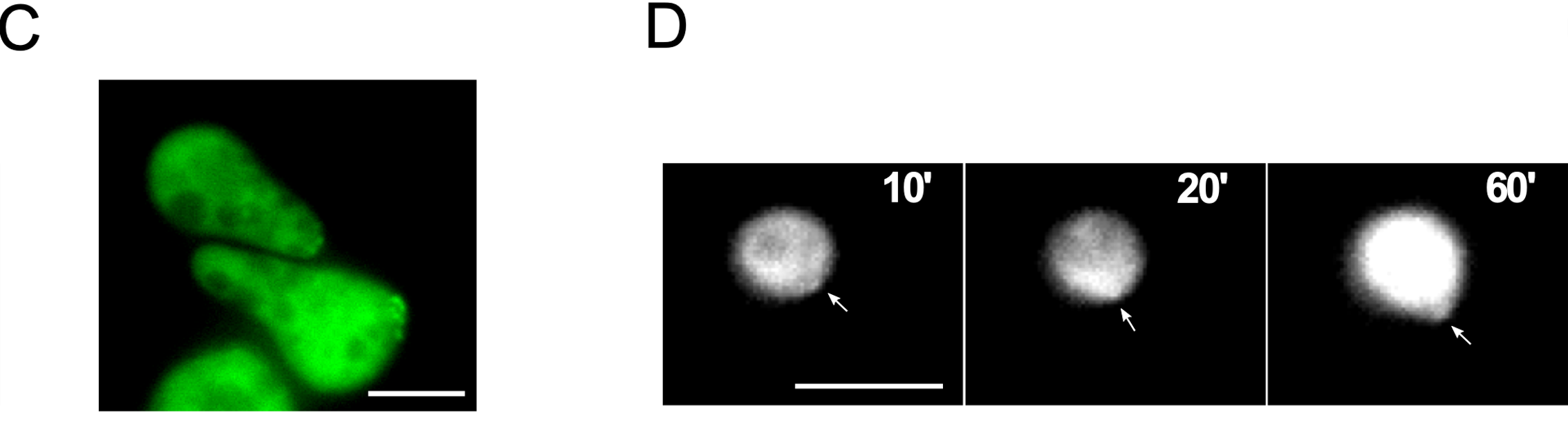


**FIGURE S1**: Prolong pheromone treatment causes yeast cells to form a second shmoo with Ste50 localization at the tip. Yeast strain transformed with wild type Ste50 on a CEN plasmid and grown under 2μM pheromone. Cells stimulated for 3-4hrs show a second shmoo (A), 1 and 2 designates 1^st^ and 2^nd^ shmoo respectively. Percentage of second shmoo at indicated time of pheromone stimulations (B). Tiny spherical particles on the cell cortex after the first shmoo (C). Cortical Ste50 patch at 10 min (D). Bar 5μm.
