# Supplementary material for: Polarization and cell-fate decision facilitated by the adaptor Ste50p in Saccharomyces cerevisiae
Source: PLoS One. 2022 Dec 20;17(12):e0278614. doi: 10.1371/journal.pone.0278614 (PMC9767377; doi:10.1371/journal.pone.0278614)
Supplement: S2 Fig — (DOCX) [file pone.0278614.s005.docx]

**S2 Figure**

**FIGURE S2:** Transcriptional activation of Ste50 RA domain mutant R296G. Yeast strain

was transformed with different alleles of Ste50, WT, RA deletion and mutant R296G. Cells were grown to late exponential phase and stimulated with 2μM alpha-factor for 4h. Promoter reporter activity was measured by Fus1-LacZ activity. Beta-galactosidase activity was measured calorimetrically as describe (Wu, *et al*., 1999; Tatebayashi *et al*., 2006).
