# Supplementary material for: Polarization and cell-fate decision facilitated by the adaptor Ste50p in Saccharomyces cerevisiae
Source: PLoS One. 2022 Dec 20;17(12):e0278614. doi: 10.1371/journal.pone.0278614 (PMC9767377; doi:10.1371/journal.pone.0278614)
Supplement: S3 Fig — (DOCX) [file pone.0278614.s006.docx]

**S3 Figure**

**FIGURE S3**: Ste50 retracts from the shmoo after shmoo maturation. Single cell analysis after time-lapse microscopy of yeast strain YCW1886 treated with pheromone. Ste50 is in the 1^st^ shmoo and moves into the 2^nd^ shmoo while retracting from the first shmoo (1-6) with time, as indicated. Bar 5μm.
