# Supplementary material for: Polarization and cell-fate decision facilitated by the adaptor Ste50p in Saccharomyces cerevisiae
Source: PLoS One. 2022 Dec 20;17(12):e0278614. doi: 10.1371/journal.pone.0278614 (PMC9767377; doi:10.1371/journal.pone.0278614)
Supplement: S4 Fig — (DOCX) [file pone.0278614.s007.docx]

**S4 Figure**

**FIGURE S4**: Polarization is propelled by increased Ste50 gene expression. Yeast cells expressing Ste50-GFP were treated with 2μM α-Factor and followed by time-lapse microscopy for at least 8hrs. Shmoo forming cell showing a surge in Ste50 expression at G1 phase of the cell cycle (mother-daughter separated at frame 3) (A & D), and their quantified GFP (B & E), and positive correlation between fluorescence and shmoo growth of cell in A & D (C & F); Pearson r =0.9800, p<0.0001; r=0.9715, p<0.0001.
